# Medical Staff of the Army

**Published:** 1898-07

**Authors:** 


**Monthly Review of Medicine and Surgery.**

*EDITORS:*

THOMAS LOTHROP, M. D. - - WM. WARREN POTTER, M. D.

All communications, whether of a literary or business nature, should be addressed to the managing editor: 284 FRANKLIN STREET, BUFFALO, N. Y.

VOL. XXXVII. JULY, 1898. NO. 12

### MEDICAL STAFF OF THE ARMY.

JUST at this time when so many of our citizens are entering the army as volunteers, there is no more absorbing question than that pertaining to their care in case of sickness or wound. Nothing is more comforting to their friends at home than the assurance that they will be skilfully and kindly cared for should dire misfortune overtake any of the "boys," as they are familiarly called, in the direction named. There are some—we hope not many—who believe or fear that a sick or wounded soldier often is treated with indifference, not to say with rudeness or even neglect. As a matter of fact the very reverse is what happens.

In the first place the government is very cautious in the selection of its medical officers for the army. They are subjected to rigid examination before appointment and they are carefully watched afterward. If any turn out to be incompetent they are promptly detected and speedily removed from authority. The chief medical officers of army corps or divisions thus far appointed are mainly taken from the regular army. Those already chosen from civil life are, for the most part, men of reputation, many having had military experience in the civil war or with the militia.

Coming now to the regimental medical officers who stand in the relation of family physicians to the troops, we may point to those serving with our 65th regiment as an example. Dr. A. H. Briggs, the surgeon, is one of our best physicians, a man of skill, judgment and kindness of heart. His two assistants, Drs. Mead and Ruffner, are young men of energy who have been well trained and will assuredly become faithful coadjutors of Surgeon Briggs. No father, mother, or near relative or friend need fear any neglect of their kinsmen by these men.

We are here reminded of a parallel that happened when Buffalo's first contribution went to the civil war. One of our foremost physicians, Dr. Charles H. Wilcox, became surgeon of the 21st Regiment and no man could have been more faithful to his trust than he. History, we believe, is repeating itself in the case of Dr. Briggs.

It is quite probable that the surgeons and assistant surgeons of other regiments in this and other states have been selected with quite as much care as those who have been appointed to the Buffalo regiment; hence, we repeat, there is no cause for anxiety with reference to the professional care of our soldiers. The medical care of soldiers is a daily necessity, hence regimental medical officers should be competent physicians, skilled in hygiene, preventive medicine, diagnosis, therapeutics and everything that pertains to the practice of medical science. Their surgical skill is of less importance, because it is only occasionally required, whereas as physicians they are constantly employed. Operating surgeons will be appointed to do that work when the necessity arises and those will be selected to perform that duty who display an aptitude for it.

In the care of sick and wounded our government supplies material with a most lavish hand. Every known necessity, not to say comfort and convenience is provided for the treatment of the sick and the care of the wounded. Field hospitals of divisions will be the campaign unit, and these will be administered by men who have executive capacity and judgment of a high order. In the civil war regimental hospitals proved inadequate and were abandoned after the first year, when division hospitals were substituted. Profiting by this experience a similar order has been issued by the surgeon-general. These hospitals will be supplied with blankets, dressings, stimulants, anesthetics, food, in short everything needed for an emergency to ensure prompt attention to the disabled soldiers. Ambulances, hospital ships and hospital trains will be luxuriously fitted out for their transportation to more permanent hospitals.

All these hospitals and everything pertaining to the management will be subjected to frequent inspection by competent officers, whose duty it will be to see that they are kept at all times up to the highest standard of efficiency and to report defects or incompetency. Under the scientific eye and skilful hand of Surgeon-General Sternberg, who is organising and directing all this vast machinery, there need be no fear that the work will not be done. On the other hand there is every reason to believe that General Sternberg will so conduct the medical department of the army as to bring credit to the American medical profession from which has been chosen the finest army medical staff the world has yet seen.

